# Identify Key Determinants of Contraceptive Use for Sexually Active Young People: A Hybrid Ensemble of Machine Learning Methods

**DOI:** 10.3390/children8110968

**Published:** 2021-10-26

**Authors:** Zongchao Liu, Zhi Lin, Wenzhen Cao, Rui Li, Lilong Liu, Hanbin Wu, Kun Tang

**Affiliations:** 1 Vanke School of Public Health, Tsinghua University, Zhongguancun North Street, Haidian District, Beijing 100084, China; zl2860@cumc.columbia.edu (Z.L.); 1710306219@pku.edu.cn (Z.L.); hanbury@link.cuhk.edu.hk (H.W.); 2 Department of Biostatistics, Columbia University Irving Medical Center, New York, NY 10032, USA; 3 School of Public Health, Peking University, Beijing 100083, China; 4 Shantou University Medical College, No. 22 Xinling Road, Shantou 515041, China; caowz@pku.edu.cn; 5 School of Public Health, Shantou University, No. 243 Daxue Road, Shantou 515063, China; 6 Department of Surgery, Washington University School of Medicine, St. Louis, MO 63130, USA; lirui@wustl.edu; 7 Department of Pharmacology, School of Basic Medical Sciences, Wuhan University, Wuhan 430071, China; liulilong@whu.edu.cn

**Keywords:** public health, contraception, machine learning, young people

## Abstract

Sexually active young people face an increasing public health burden of unintended pregnancies and sexually transmitted diseases due to improper contraception. However, environmental and social factors related to young people’s contraception remain unclear. To identify the key factors, we applied ensemble machine learning methods to the data of 12,280 heterosexual Chinese college students who reported sexual intercourse experience in the National College Student Survey on Sexual and Reproductive Health in 2020 (NCSS-SRH 2020). In the order of variable importance, convenient access to contraceptives, certain attitudes towards sex, sexual health knowledge level, being an only-child, and purchasing a bachelor’s or master’s degree were positively associated with a high frequency of contraceptive use. In contrast, smoking, free access to contraceptives, a specific attitude towards marriage, and negotiation with a sexual partner were negatively associated with a higher frequency of contraceptive use. Our analysis provides insights into young people’s contraceptive use under a typically conservative culture of sexuality. Compared to previous studies, we thoroughly investigated internal and external factors that might impact young people’s decision on contraception while having sex. Under a conservative culture of sexuality, the effects of the external factors on young people’s contraception may outweigh those of the internal factors.

## 1. Introduction

With abortion as the leading cause of death among young girls (aged 10–24 years), unintended pregnancy brings a heavy burden globally [1]. In each year, 38% of an estimated 210 million pregnancies worldwide are unintended, of which 22% result in abortion [2]. Unintended pregnancies posed severe physical and psychological health issues in young girls, causing significant social burdens [3]. Regarding the negative impacts, modern contraceptive methods have been proved to be the most effective way for young people to prevent unintended pregnancies and their related complications [4]. Nowadays, young people face various options of modern contraceptive methods, including condoms, oral contraceptives, injectable contraceptives, emergency contraceptive pills, and intrauterine contraceptive devices (IUD) [5]. Of these methods, using condoms has been regarded as the most convenient and efficient.

Available evidence shows that sexually active young people usually do not expect pregnancy [6]. They are supposed to have positive attitudes and good awareness towards contraception, but their actual contraceptive use during sexual intercourse is not ideal, especially in developing countries [4,7], where they face tremendous obstacles in obtaining or practicing modern methods of contraception [3,8,9,10]. The gap between the actual use and awareness of contraceptive methods corresponds to a complex interaction by the factors related to contraceptive behaviors. Previous studies have revealed significant associations between contraceptive use and a number of internal factors like awareness, attitudes, and self-experiences [3,6,7,9,11]. Compared to these internal factors, more and more external (i.e., environmental) factors were also verified as influential on young people’s behaviors of sexual health. The socioeconomic status, sex education, family background, and accessibility of contraceptives have been reported to be associated with young people’s contraceptive use [3,12]. In addition, mental health history and lifestyles were shown as associated with contraceptive experience of young people [12,13,14].

Before this study, the above factors were investigated fragmentedly across different studies. Therefore, one was not able to differentiate the relative importance of one factor from another. Herein, based on the nationwide study, we applied machine learning methods to identify and compare the importance of a complete spectrum of internal and external factors. To see how the important factors affected young people’s contraceptive behaviors, we further evaluated their effects under a conservative culture of sexuality.

## 2. Materials and Methods

### 2.1. Data Source

The study employed the data from a large web-based survey, the National College Student Survey on Sexual and Reproductive Health in 2020 (NCSS-SRH 2020) sponsored by the China Family Planning Association (CFPA). The survey was conducted among approximately 0.19% of Chinese college students from November 2019 to February 2020. Using multistage sampling, 241 higher education institutions were selected after balancing the population density and different levels of educational institutions in China. The unique web link for the electronic questionnaire was distributed to voluntary participants through contact persons in each institution where the survey was conducted. A total of 55,757 respondents completed questionnaires. Of those, 1177 (2%) responses were eliminated because the respondent either did not properly complete the attention check questions, endorse the informed consent, or was outside the age range of college students. Valid participants were 54,580 youth (65.5% female and 77.6% heterosexual) from Eastern (52.3%), Central (24.4%), and Western (23.3%) China. Before completing the survey, each participant provided informed consent. Of the valid participants, 12,280 heterosexual participants who reported sexual intercourse experience were identified as high-risk population of unintended pregnancy and finally included for analyses.

### 2.2. Primary Outcome and Covariates

The study’s primary outcome was the frequency of contraceptive use (FCU). The FCU was initially computed as a 5—level ordinal categorical variable (1—Never; 2—Seldom; 3—About half of the time; 4—Usually; 5—Always) in the survey and was re-coded as a composite score ranging from 1 to 3 (1—Level 1: Never & Seldom; 2—Level 2: About half of the time & Usually; 3—Level 3: Always) for a more parsimonious and informative indication in analyses. Referring to previous related studies [3,12,13,14], we included 25 potential variables (covariates) across eight domains: education-related factors, socio-economics, attitudes towards sexuality, sexual health knowledge, sexual and mental health history, contraceptive accessibility, family-related factors, and lifestyles. The factors associated with FCU were later selected from the variables of these domains. Details about the variables were included in the Supplementary Materials.

### 2.3. General Workflow

**Figure 1:**
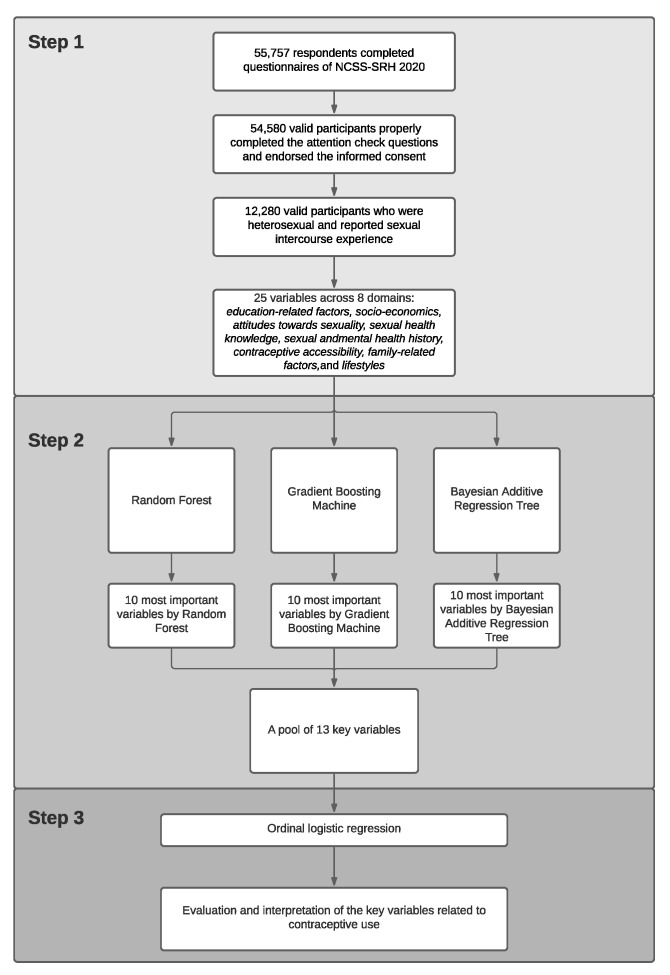
General workflow for identification and interpretation of the determinants associated with the frequency of contraceptive use (FCU) among young people. The general workflow contained 3 steps. Step 1 was for data preparation; Step 2 was for model training and variable selection; and Step 3 was for quantitative evaluation on key determinants by proportional odds model.

The variable ranking and selection processes for key variable identification were preceded by building three types of ensemble machine learning models: Random Forest (RF), Gradient Boosting Decision Trees (GBDT), and Bayesian additive regression trees (BART). The general workflow of this study consisted of three steps (Figure 1). In Step 1, sample selection, preparation, and feature engineering were all conducted. In Step 2, the prepared data were split into a training set and a test set with the split ratio of 7:3. The training data with the 25 variables were then used to train on three machine-learning models (RF-full, GBDT-full, and BART-full). Rooted mean square error (RMSE) was applied to evaluate model performance, and the models were tuned by 10-fold cross-validation. After evaluating the trained models’ performance, we selected the top 10 most important variables from each of these models and combined them as a new pool of “key” variables. To further ensure the efficacy of these variables for predicting FCU, this set of key variables was again used to train on three new nested models (RF-10, GBDT-10, and BART-10). The nested models’ performance was then compared with the full models’ one. In Step 3, after ensuring the effectiveness of the “key” variables, we conducted an ordinal logistic regression model to quantitatively evaluate and interpret the effects of each key variable on FCU.

### 2.4. Criteria of Variable Selection

The variables were ranked according to the variable importance obtained from trained models. For RF and GBDT, the variable importance was indicated by the reduction of squared error attributable to each variable [15,16]. For BART, the variable importance was represented by the variable inclusion proportion, the proportion of times that each variable was selected as a splitting rule divided by the total number of splitting rules in building the model [17,18]. The rank of these variables would be deemed as valid only when the source model performed reasonably well. The selected “important” variables represented most of the predictive efficacy for inferencing one’s contraceptive behaviors.

### 2.5. Evaluating the Variable-Outcome Relationships via Ordinal Logistic Regression

To tackle the poor interpretability of the applied machine learning methods and strengthen the study findings, we further fitted an ordinal logistic regression model to describe the effects of each selected key factor on FCU. The selected key factors were from the combined set of key variables identified by the three full models (RF-full, GBDT-full, and BART-full). Odds ratios (ORs) with 95% confidence intervals (95% CIs) were reported. Across the study, statistical significance thresholds were all set to be 0.05 with two sides.

## 3. Results

### 3.1. Model Performance and Identified Determinants

Table 1 displayed the operational parameters, the model performance on the training and test sets, and the selected 10 most important variables associated with FCU for the three types of models. Among the full models, RF-full had the lowest RMSE with 0.7125 on the training set, and BART-full had the lowest RMSE with 0.7720 on the test set. The selected variables from each model were combined as a new pool of “key” variables, containing a total of 13 variables. As shown in Table 1, we used the set of “key” variables to train on three new nested models (RF-10, GBDT-10, and BART-10). Among the nested models, RF-10 had the lowest RMSE with 0.7171 on the training set, and BART-10 had the lowest RMSE with 0.7741 on the test set.

### 3.2. Ordinal Logistic Regression Analysis

An ordinal logistic regression model stratified by sex was fitted between FCU and the selected variables (Table 2). The presented OR accounted for the effect of a specific variable on the odds ratio for higher level(s) of FCU versus the lower level(s).

Among males and females, a higher sexual health knowledge level was positively associated with FCU (male OR: 1.10, 95% CI 1.06–1.14; female OR: 1.12, 95% CI 1.07–1.16). Participants’ attitudes towards sexuality also impacted FCU. Given the answers (all binary responses indicated by “Yes” or “No”) to 12 attitude-related questions in the survey, participants were clustered into three sub-groups (attitude cluster 1, cluster 2, and cluster 3) by hierarchical clustering (Supplementary Materials). Compared to attitude cluster 1, cluster 2 and 3 were both positively associated with the FCU (male *cluster 2* OR: 2.02, 95% CI 1.64–2.48; male *cluster 3* OR: 1.87, 95% CI 1.53–2.27; female *cluster 2* OR: 1.48, 95% CI 1.22–1.81; female *cluster 3* OR: 1.37, 95% CI 1.11–1.70). In terms of Individual factors, being an only-child presented positive association with FCU (male OR: 1.46, 95% CI 1.23–1.72; female OR: 1.38, 95% CI 1.19–1.61). We further found that pursuing a bachelor’s degree was positively associated with FCU among males (OR: 1.51, 95% CI 1.25–1.82), and pursuing a master’s degree was positively associated with FCU among females (OR: 1.53, 95% CI 1.15–2.03). Regarding the contraceptive accessibility, convenient access to contraceptives was significantly related to a higher FCU among males and females (male OR: 1.47, 95% CI 1.19–1.81; female OR: 1.69, 95% CI 1.41–2.04). Among females, free access to contraceptives was related to a lower FCU (OR: 0.77, 95% CI 0.61–0.97). The results also implied that negotiation on contraception with a sexual partner might lead to a lower FCU, as *contraception decided together* (male OR: 0.75, 95% CI 0.63–0.90; female OR: 0.82, 95% CI 0.67–0.99) and *contraception only decided by the sexual partner* (male OR: 0.34, 95% CI 0.26–0.43; female OR: 0.32, 95% CI 0.24–0.42) were all negatively associated with FCU among males and females.

## 4. Discussion

By defining FCU as the primary outcome, the study adopted ensemble machine learning methods to identify key factors that might significantly impact young people’s contraceptive behaviors. We further evaluated the associations between the identified factors and FCU. As the times of sexual activities increase, the cumulative risk of unintended pregnancy also increases if one does not take proper actions for contraception during sexual intercourse experience. Therefore, it is more significant to look into one’s actual times (i.e., frequency) of contraceptive use than only documenting whether one has ever taken contraceptive actions. Before this study, however, most research treated contraceptive behaviors as a “one-time” action and encoded them as a binary indicator for analysis, playing down the importance of evaluating the total times of contraception across all of a person’s sexual intercourse experiences. Also, many previous studies emphasized on interpreting the association between contraceptive behaviors and internal factors (attitudes, knowledge, behaviors, etc.), ignoring several external or environmental factors that might also be determinant of the behavioral pattern. This might lead to a problem of interpreting the actual situation of young people’s contraception: why do they have poor actions in contraception, even though most of them hold positive attitudes and a high level of knowledge towards contraception [14]? To properly tackle the problems above, we applied machine-learning methods to identify and validate the internal and external factors related to contraceptive behaviors. We used FCU, a multi-level variable, as the primary outcome to depict one’s contraceptive behaviors more specifically.

Our study shows that negotiation on contraception with a sexual partner may ultimately lead to a decreased FCU during sexual activities among males and females. Such negative impact on FCU might be largely attributed to males, as previous studies have reported that males’ attitudes largely influence females’ decision in contraceptive use during sexual activities, either in positive or negative directions under different scenarios [19,20,21]. In addition to males, our findings further imply that females also tend to bring down the FCU while having sex with males. Further qualitative studies are required to investigate what leads to a reduced frequency of contraception after negotiation between sexual partners and to validate whether males or females are more influential on contraception decisions.

Our study indicates a positive relationship between convenient access to contraceptives and FCU during sexual activities among males and females. The relationship is consistent with previous finding that the rate of contraceptive use was positively correlated to the accessibility of contraceptives [22,23,24,25]. Additionally, in a study conducted on Chinese college students, 61.6% of the participants reported that they would prepare a condom before having sex, while 20.1% of the participants reported that they would feel nervous or embarrassed to buy condoms [12]. These all imply that improving accessibility of contraceptives is reasonable to protect young people, though psychological factors that may influence young people’s buying behaviors should also be taken into account. Many universities in China have launched projects to provide free condoms for college students, aiming at preventing the students from unintended pregnancy and STD [26]. Under a conservative culture of sexuality, such actions worked well in tackling students’ embarrassment of buying condoms and thus improved actual FCU among college students.

Apart from the convenient access to contraceptives, our study finds a negative correlation between the free access to contraceptives and the frequency of contraceptive use among females. One possible explanation for this situation can be conceived under a conservative culture of sexuality: Young people can easily get free access to contraceptives in more public places than a private environment, and being exposed to public places might make young females feel embarrassed and less willing to access contraceptives in such an environment. This assumption was strengthened by a previous study showing that private access to condoms would increase uptake [25].

Our study highlights the significance of internal factors in a way different from previous studies, showing that attitude towards sexuality does impact FCU. By performing a hierarchical clustering analysis on the participants’ attitudes towards 12 specific sex-related questions, we clustered the participants into three sub-groups (*attitude-1*, *attitude-2*, *and attitude-3*). The clustering process was independent of FCU, allowing us to explore the heterogeneity and homogeneity of the complex attitudes among the three sub-groups without incorporating the outcome variable. The association between the sub-groups and FCU was later validated by our machine-learning models and quantified by the ordinal logistic regression. Our findings show that participants who belonged to the attitude cluster 2 and cluster 3 tend to have higher FCU than participants in the attitude cluster 1. A majority (78%) of the participants in our study belonged to cluster 2 and cluster 3, implying an overall tendency of high FCU. However, this contrasts with a previous claim that a large proportion of college students were holding positive attitudes towards contraception, even though the actual situation of contraceptive use among them was not ideal [6]. A potential explanation for this may be that there exist some other external factors with strong impacts on FCU. Regarding participants’ willingness and attitudes towards marriage, we identified a group of young people with low FCU and high risk of unintended pregnancy or STDs: Those females who did not want to get married but planned to live with their boyfriend for a long time tended to have lower FCU. It is worthwhile to think of improving their awareness of contraception.

The present study shows that an only-child may have a higher FCU compared to those who grew up with siblings. However, there has been a lack of in-depth exploration and discussion on the association between an only-child and the contraception details. Referring to the recent patterns of economics and policy development in China, it is conceivable that only-children in China were more likely born in towns and received better sex education that raised their awareness of contraception [27]. In terms of the participants’ education level, our study shows that young males pursuing a bachelor’s or young females pursuing a master’s degree tend to have higher FCU. Previous studies can partly explain this phenomenon: with a higher degree of education, students may have more opportunities to learn sexual knowledge and raise awareness of contraception [28,29].

Compared to previous studies, the present study has several advantages. First, we used FCU, a multi-level ordinal variable as the outcome to indicate contraceptive behaviors. Compared to most previous studies that used a binary outcome to indicate contraception, our study is more flexible in depicting the relationship between FCU and several external factors, given more information contained by FCU than the binary indicators. Second, the sample size (*n* = 12,280) for our study was adequately large, allowing us to train stable machine-learning models. Third, the methods we applied were well suited to our research interest—to select a set of key external factors related to FCU, and have worked efficiently in other large-scaled survey studies [30,31]. To increase the power for detecting significant external factors, we combined the three different sets of selected variables from RF, GBDT, and BART as a pool of key variables. For each model, the variable selection process was independent. The results were robust as the selected variables across the three different models had great intersections and the performance of the new models using only the key predictors was similar to the full models. Finally, by adopting ordinal logistic regression, we successfully quantified and interpreted the effects of the selected key variables associated with FCU. The selected variables also included internal factors like participants’ attitudes and sexual knowledge, which strongly coincided with previous findings.

However, the present study has some potential limitations. First, although we had a large sample size, the proportion of the participants with low FCU was relatively small. Such unbalanced data might result in inadequate training of the machine learning models. Second, although the FCU was originally ordinal and discrete, different levels of the FCU were not guaranteed to be mutually exclusive. Therefore, while training machine-learning models, rather than treating FCU as a categorical variable, we treated it as a continuous variable. This caused all three models to work on a regression task rather than a relatively simple classification task, thus inducing relatively high prediction errors acceptable for exploration. Third, though hierarchical clustering helped reduce the dimensions of the attitudes-related variables in our study, we could not precisely illustrate the exact meaning of the particular attitudes for each sub-group. It is encouraged to further investigate the actual attitudes towards contraception or other topics for each sub-group. Fourth, according to the results from ordinal logistic regression, not all the selected key variables’ coefficients were statistically significant in the model. This might be due to the difference in the optimization process of the ensemble machine-learning models. It is challenging to ensure that all the selected variables were interpretable while simultaneously being significantly associated with the outcome of interest. Finally, we have not included sexual minority groups for analysis as they may not involve unintended pregnancy in certain circumstances.

## 5. Conclusions

Several key external and internal factors related to young people’s contraceptive behaviors were identified by applying ensemble machine-learning models. Convenient access to contraceptives, certain attitudes towards sexuality, higher level of sexual health knowledge, being an only-child, and purchasing a bachelor’s or master’s degree were positively associated with the frequency of contraceptive use. In contrast, depression, smoking history, free access to contraceptives, specific attitude towards marriage, and negotiation with a sexual partner were negatively associated with the frequency of contraceptive use. The results provide insights into interventions to prevent unintended pregnancies in young people.

## Figures and Tables

**Table 1:** Model performance and identified key variables.

Machine Learning Methods ^a^	Tuning Parameters	Training RMSE ^b^ (Full Models)	Test RMSE ^b^ (Full Models)	Training RMSE ^b^—(Models with Top 10 Important Variables)	Test RMSE ^b^—(Models with Top 10 Important Variables)	Top 10 Important Variables
RF	Number of trees Node size Number of predictors	0.7125	0.775	0.7171	0.7796	Negotiation, SRH-quiz score, Depression, Age, Convenient accessibility, Educational level, Enrollment year, Attitude towards sexuality, Smoking, Attitudes towards marriage
GBM	Number of trees Node size Number of predictors Shrinkage rate	0.731	0.7753	0.7412	0.7752	Negotiation, SRH-quiz score, Convenient accessibility, Age, Enrollment year, Depression, Attitude towards sexuality, Educational level, Smoking, Romantic relationship
BART	Number of trees Quantile of the prior on the error variance at which the data-based estimate is placed Degrees of freedom for the inverse *χ*^2^ prior	0.7562	0.772	0.7566	0.7741	SRH-quiz score, Enrollment year, Attitude towards sexuality, Negotiation, Educational level, Age, Marriage, Free access, Alcohol drinking, Only-child

Note: The table shows the operational parameters for the three types of models (including full models and nested models with top 10 important variables), the model performance on the training and test sets, and the selected 10 most important variables associated with FCU. ^a^ The methods include: RF (Random forest); GBM (Gradient boosting machine); BART (Bayesian additive regression trees); ^b^ RMSE: Rooted mean square error.

**Table 2:** Ordinal logistic regression to quantify the effects of the identified determinants on the frequency of contraceptive use (FCU).

	Male			Female		
	OR	95% CI	*p*-Value	OR	95% CI	*p*-Value
**Negotiation on contraception with sexual partners or not**						
Reference: self-decision without the involvement of the sexual partner	-	-	-	-	-	-
Both (the participant with the sexual partner) involved in decision	0.75	(0.63, 0.90)	0.002 *	0.82	(0.67, 0.99)	0.044 *
Only decided by the sexual partner	0.34	(0.26, 0.43)	<0.001 *	0.32	(0.24, 0.42)	<0.001 *
Random decision dependent on the surrounding environment	0.40	(0.27, 0.60)	<0.001 *	0.90	(0.70, 1.16)	0.419
**SRH ^a^ quiz score**	1.10	(1.06, 1.14)	<0.001 *	1.12	(1.07, 1.16)	<0.001 *
**CES-D-10 ^b^ score**	1.00	(0.98, 1.01)	0.573	0.99	(0.97, 1.00)	0.013 *
**Attitude cluster (attitudes towards sexuality)**						
Reference: Cluster 1	-	-	-	-	-	-
Cluster 2	2.02	(1.64, 2.48)	<0.001 *	1.48	(1.22, 1.81)	<0.001 *
Cluster 3	1.87	(1.53, 2.27)	<0.001 *	1.37	(1.11, 1.70)	0.004 *
**Enrollment year**	1.00	(0.94, 1.05)	0.855	0.99	(0.94, 1.03)	0.587
**Attitude towards marriage**						
Reference: Must get married anyway	-	-	-	-	-	-
Get married if there is a suitable partner, otherwise it is OK not to get married	1.08	(0.91, 1.29)	0.385	1.25	(1.00, 1.57)	0.045 *
Do not expect to get married, but can live with girl/boyfriends for a long time	1.18	(0.84, 1.64)	0.341	0.70	(0.51, 0.95)	0.023 *
Do not want to get married, would rather be single all the time	0.75	(0.39, 1.44)	0.391	0.85	(0.43, 1.68)	0.634
**Convenient Access to contraceptives**	1.47	(1.19, 1.81)	<0.001 *	1.69	(1.41, 2.04)	<0.001 *
**Only-child**	1.46	(1.23, 1.72)	<0.001 *	1.38	(1.19, 1.61)	<0.001 *
**Free access to contraceptives**	0.85	(0.7, 1.04)	0.118	0.77	(0.61, 0.97)	0.027 *
**Degree**						
Reference: Associate	-	-	-	-	-	-
Bachelor’s	1.51	(1.25, 1.82)	<0.001 *	1.24	(1.04, 1.49)	0.016 *
Master’s	1.25	(0.88, 1.78)	0.208	1.53	(1.15, 2.03)	0.004 *
Ph.D.	1.32	(0.74, 2.36)	0.353	0.85	(0.41, 1.77)	0.661
**Smoking**						
Reference: Never smoke	-	-	-	-	-	-
Used to smoke but already gave up	0.80	(0.63, 1.02)	0.072	0.71	(0.58, 0.89)	0.002 *
Currently Smoke	0.81	(0.66, 0.98)	0.033 *	0.63	(0.49, 0.80)	<0.001 *
**Romantic relationship**						
Reference: Never have a boyfriend/girlfriend	-	-	-	-	-	-
Have a boyfriend/girlfriend before	0.96	(0.63, 1.47)	0.863	0.79	(0.38, 1.64)	0.534
Currently have a boyfriend/girlfriend (or married)	1.27	(0.83, 1.93)	0.275	0.93	(0.45, 1.91)	0.843
**Alcohol drinking**	1.04	(0.86, 1.25)	0.695	0.91	(0.78, 1.07)	0.252

Note: * Statistical Significance, *p* < 0.05 (two sided). Variable names are marked in bold. ^a^ SRH quiz: the Sexual Reproductive Health quiz embedded in the survey (Supplementary Materials). The quiz contained nine questions and included topics on contraception, HIV/AIDS, pregnancy/abortion. The score for the quiz ranged from 0–9, corresponding to the number of correct answers attained; ^b^ CES-D-10: the 10-item Centre for Epidemiological Studies Depression Scale, a self-report depression scale for research in the general population.

## Data Availability

The data are not publicly available due to ethical restrictions.

## Supplementary Materials

The following are available online at https://www.mdpi.com/article/10.3390/children8110968/s1, Table S1: Included covariates for identifying key determinants associated with young people’s frequency of contraceptive use (FCU); Table S2: The list of survey questions regarding attitudes towards sexuality; Table S3: The list of questions in the SRH quiz; Table S4: Types of the sexual harassment experience asked in the survey.
