# Supplementary material for: Identify Key Determinants of Contraceptive Use for Sexually Active Young People: A Hybrid Ensemble of Machine Learning Methods
Source: Children (Basel). 2021 Oct 26;8(11):968. doi: 10.3390/children8110968 (PMC8622295; doi:10.3390/children8110968)
Supplement: Supplementary file 1 [file children-08-00968-s001.zip › children-1395986-supplementary.pdf]

Supplementary Materials

Detailed information about the included variables

We included 25 potential variables across 8 domains (**Table 1**): *education-related factors, socio-economics, attitudes towards sexuality, sexual health knowledge, sexual and mental health history, contraceptive accessibility, family-related factors,* and *lifestyles*. Most of the included variables were continuous or categorical variables. Notably, for measuring the participants' attitudes towards sexuality, the survey asked 12 designed questions (**Table 2**). A 12-dimension vector containing the responses to these 12 questions was obtained for each participant. Later, a hierarchical clustering analysis was applied on the vectors to assign the participants into 3 sub-groups, using Euclidean distance as the distance metric and adopting the complete-linkage criteria. The label attained by clustering analysis for each participant indicated the sub-group that the participant was belonged to, and the sub-groups with different labels were assumed to be different (holding different mixtures of attitudes) from each other. The label was later used as a predictor for model training to validate the effects of internal factors on contraceptive behaviors. Participants' sexual health knowledge levels were quantitatively measured by a short Sexual Reproductive Health (SRH) quiz containing 9 questions (**Table 3**). The quiz included topics on contraception, HIV/AIDS, pregnancy/abortion. The score for the quiz ranged from 0 to 9, corresponding to the number of correct answers attained. In the domain of *sexual and mental health history*, the depression status for each participant was measured by the 10-item Centre for Epidemiological Studies Depression Scale (CES-D-10), a self-report depression scale for research in the general population. There was also an indicator encoded as a binary variable documenting each participant's sexual harassment history (0 - no harassment; 1 - experienced harassment). This indicator was transformed based on a series of questions asking about sexual harassment experience (**Table 4**). A participant was considered to be sexually harassed before if any confirmative answer to such questions was reported.

Table S1. Included covariates for identifying key determinants associated with young people's frequency of contraceptive use (FCU)

Variable name	Description	Original/ Composite[a]	Number of source questions in the survey	Variable type	Value/Range
Education-related factors					
School location	The school's location	Original	1	Categorical	West - 1; Middle - 2; East - 3
School type	The school type (university or vocational school)	Original	1	Categorical	University (College) - 0; Vocational School - 1
Educational level (degree)	The participants' educational level	Original	1	Categorical (ordinal)	Associate - 1;<br>Bachelor - 2;<br>Master - 3;<br>Ph.D. - 4
Undergraduate/Graduate student	Whether the participant is an undergraduate or graduate student	Original	1	Categorical	Graduate - 0; Undergraduate - 1
Enrollment year	The participant's enrollment year	Original	1	Continuous	-4 ~ 4 (integer) corresponding to enrollment year in 2011 ~ 2019
Socio-economics					
Sex	Sex of the participant	Original	1	Categorical	Female - 0; Male - 1
Age	Age of the participant	Original	1	Continuous	15 ~ 29 (integer) years old
Attitude towards marriage	The participant's attitudes towards marriage	Original	1	Categorical	Must get married anyway - 1;<br>Get married if there is a suitable partner, otherwise it is OK not to get married - 2;<br>Do not want to get married, but can live with girl/boyfriends for a long time - 3;<br>Do not want to get married, would rather be single all the time - 4

Variable name	Description	Original/ Composite[a]	Number of source questions in the survey	Variable type	Value/Range
Monthly expenditure	The participant's monthly expenditure	Original	1	Categorical (ordinal)	< 1500 CNY - 1;<br>1500 ~ 2500 CNY - 2;<br>>2500 CNY - 3
Romantic Relationship	Whether the participant had a romantic relationship	Original	1	Categorical	Never have a boyfriend/girlfriend - 1;<br>Have a boyfriend/girlfriend before - 2;<br>Currently have a boyfriend/girlfriend (or married) - 3
Attitudes towards sexuality					
Attitude towards sexuality	The participant's attitudes towards sexuality	Composite	12	Categorical	Cluster 1 - 1;<br>Cluster 2 - 2;<br>Cluster 3 - 3
Sexual health knowledge					
SRH-quiz[b] score	The score obtained by the participant during the SRH quiz of the survey	Composite	9	Continuous	0 ~ 9 (integer) corresponding to the number of correct answers to questions in the SRH quiz
Sexual and mental health history					
Sexual harassment	Whether the participant experienced sexual harassment	Composite	7	Categorical	Yes - 1; No - 0
STD	Whether the participant had sexually transmitted diseases	Original	1	Categorical	Yes - 1; No - 0

Variable name	Description	Original/ Composite[a]	Number of source questions in the survey	Variable type	Value/Range
Depression status	Whether the participant was depressed according to the CES-D-10[c] evaluation	Composite	1	Continuous	Values were the scores of CES-D-10
Contraceptive accessibility					
Convenient Accessibility	Whether it is convenient for the participant to access contraceptives	Original	1	Categorical	Yes - 1; No - 0
Free access	Whether the participant had free access to contraceptives	Original	1	Categorical	Yes - 1; No - 0
Negotiation	Whether the participant negotiated on contraception with the sexual partner and who finally made a decision to contraception	Original	1	Categorical	self-decision without the involvement of the sexual partner - 1; Both (the participant with the sexual partner) involved in decision - 2; Only decided by the sexual partner- 3; Random decision dependent on the surrounding environment - 4
Family-related factors					
Father’s educational level	The participant's father's educational level	Original	1	Categorical (ordinal)	Below bachelor’s degree - 1; bachelor’s degree or equivalent - 2; Above bachelor’s degree - 3

Variable name	Description	Original/ Composite[a]	Number of source questions in the survey	Variable type	Value/Range
Mother's educational level	The participant's mother's educational level	Original	1	Categorical (ordinal)	Below bachelor's degree - 1; bachelor's degree or equivalent - 2; Above bachelor's degree - 3
Only-child	Whether the participant is an only-child	Original	1	Categorical	Yes - 1; No - 0
Hometown Area	The hometown area where the participant used to live	Original	1	Categorical	Rural - 1; Urban - 2; Suburban - 3
Divorced family	Whether the participant lived in a divorced family	Original	1	Categorical	Yes - 1; No - 0
Lifestyles					
Alcohol drinking	Whether the participant drank alcohol	Original	1	Categorical	Yes - 1; No - 0
Smoking	Whether the participant smoked	Original	1	Categorical	Never - 1; Never smoke - 1; Used to smoke but already gave up – 2; Currently Smoke - 3

Note. 25 potential variables (covariates) across 8 domains: *education-related factors, socio-economics, attitudes towards sexuality, sexual health knowledge, sexual and mental health history, contraceptive accessibility, family-related factors,* and *lifestyles*. The determinants associated with FCU were later selected from these variables.

[a] Original: the values of the variable were taken directly from the dataset without being further re-coded; Composite: the values of the variable were processed before being used for analysis.

[b] SRH quiz: Sexual Reproductive Health quiz embedded in the survey (**Supplementary material**). The quiz contained 9 questions and included topics on contraception, HIV/AIDS, pregnancy/abortion. The score for the quiz ranged from 0 to 9, corresponding to the number of correct answers attained.

[c] CES-D-10: the 10-item Centre for Epidemiological Studies Depression Scale, a self-report depression scale for research in the general population.

Table S2. The list of survey questions regarding attitudes towards sexuality

Number	Questions	Answers
**1**	Entering the sexually active period makes me feel guilty	Strongly disagree;<br>Disagree;<br>Not sure;<br>Agree;<br>Strongly agree
**2**	Sex education will lead to [earlier] sexual behaviors	
**3**	Sex education will lead to [more] sexual behaviors	
**4**	If you have sex before marriage, your married life will be happier in the future	
**5**	It is acceptable to have sex for college aged youths	
**6**	The Internet makes making friends more easily, and it also makes one-night stands more common	
**7**	You don’t necessarily have to wear a condom every time, if you don’t necessarily get “in”	
**8**	An occasional "one-night stand" is not a serious betrayal of love	
**9**	Having multiple sexual partners at the same time is acceptable	
**10**	In real society, men still prefer virgins	
**11**	Homosexuality is normal	
**12**	I am willing to go to school with classmates infected with HIV	

Table S3. The list of questions in the SRH quiz

Number	Questions	Potential answers	Correct answers
**1**	Sexual activity about 14 days before menstruation is most likely to cause pregnant		Yes
**2**	Sperm can normally survive in a woman's body for about 7 days		No
**3**	As long as the timing is accurate, extracorporeal ejaculation can effectively prevent pregnancy		No
**4**	Having sex during a "safe period" can effectively avoid pregnancy	Yes;	No
**5**	While having sex, condoms are the only way to avoid pregnancy, STDs, and AIDS	No;	Yes
**6**	Mosquito bites may be a mediator to transmit AIDS	Not sure	No
**7**	Genital herpes is a kind of sexually transmitted disease		Yes
**8**	After a woman is pregnant, her menstruation will continue for two to three months		No
**9**	Compared with ordinary abortion, painless abortion is safer		No

Table S4. Types of the sexual harassment experience asked in the survey

Number	Questions	Answers
**1**	Being laughed at by classmates due to physical development	Yes; No
**2**	Verbal sexual harassment in real life	
**3**	Verbal sexual harassment on the Internet or on mobile phones	
**4**	Forced undressing/exposure of private body parts	
**5**	Forced kissing or touching private parts	
**6**	Forced oral sex	
**7**	Forced sexual intercourse (vaginal/anus)	
